# Assessment of microcirculation on sternal wound healing after coronary artery bypass graft surgical diabetic & non-diabetic patients using laser Doppler imaging system

**DOI:** 10.1186/1749-8090-10-S1-A268

**Published:** 2015-12-16

**Authors:** Premjithlal Bhaskaran, Mohamed Aslam, Nigel J Standfield, Terence Gourlay

**Affiliations:** 1 Hammersmith Hospital, Imperial College, London, W12 0HS, UK; 2 University of Strathclyde, Glasgow, G4 0NW, UK

## Background/Introduction

In this study we propose to investigate the effect of the neovascularisation and blood flow to the sternum and surrounding tissues in diabetic & non-diabetic patients by using a laser Doppler imager. The reasons for the delayed healing are not entirely clear, but are thought to include both mechanical factors such as poor wiring of the breastbone during surgery, and biological factors related to the interruption to the blood supply to the sternum and surrounding tissues associated with the procedure.

## Aims/Objectives

To assess the role of neovascularisation in diabetic & non-diabetic following coronary artery bypass graft (CABG) surgery using Laser Doppler Imager.

## Method

Patients were divided into two groups as diabetic & non-diabetic had undergone CABG (30 patients). Sternal microcirculation measurements were taken by using a Moor LDI laser Doppler imaging system, at ten time points (pre-induction to 96 hours after bypass). The regional blood flow was estimated by measuring the Doppler shift of laser light caused by blood cells passing within the laser light field. Blood samples were taken for the analysis of number of factors.

## Results

Results: The neovascularisation and wound healing were comparatively faster in non-diabetic surgical patients than diabetic group. New vessel formation from the right internal thoracic and intercostal arteries to the left side confirmed that the vascular supply of the sternum on the left side following CABG surgery was not entirely depended upon the left internal thoracic arteries. This is due to secondary changes in diabetic patients on vascular system.

## Discussion/Conclusion

There was a formation of new vessels from right side of the sternum following the mobilization of left internal thoracic artery in CABG surgical patients. The healing process was faster in non-diabetic CABG surgical patients.

